# Deep sequencing of single *Pleurotus ostreatus* strain revealed mixed infection of seven RNA viruses

**DOI:** 10.1186/s12864-026-13166-z

**Published:** 2026-07-17

**Authors:** Jiayi Gao, Guoqiang He, Jin Kang Wei, Yong Pan, Congliang Deng, Xiju Shi, Caige Lu, Yongqiang Li

**Affiliations:** 1 Key Laboratory for Northern Urban Agriculture of Ministry of Agriculture Rural Affairs, College of Bioscience and Resource Environment, Beijing University of Agriculture, Beijing, 102206 China; 2 Beijing Agricultural Technology Extension Station, Beijing, 100029 China; 3 Science and Technology Research Center of China Customs, Beijing, 100026 China; 4 Institute of Plant Protection, Beijing Academy of Agriculture and Forestry Sciences, Beijing, 100097 China

**Keywords:** *Pleurotus ostreatus*, Deep sequencing, Virome

## Abstract

**Background:**

As a globally cultivated edible fungus, *Pleurotus ostreatus* is highly nutritious and rich in proteins, vitamins, minerals, and amino acids. However, viral infections cause significant losses in quality and yield during cultivation. In our survey, weakened growth and malformed fruiting bodies were observed in Sanbaoxiang Agricultural Technology Park, which is located in Tongzhou District of Beijing, but the associated viruses were not clear.

**Results:**

The mycelia were isolated, purified and used for ribosomal RNA-depleted total RNA deep sequencing. A total of 118,973,697 clean reads were obtained and subsequently assembled into 53,864 contigs. BLAST analysis indicated that most contigs originated from the host *P. ostreatu*s; however several contigs showed high amino acid sequence identities to known mycoviruses. Further complete genome sequencing with RT‒PCR and rapid amplification of cDNA ends for the terminal sequences revealed that mixed infection with seven RNA viruses was associated with the observed symptoms: four reported viruses, including oyster mushroom spherical virus, pleurotus ostreatus deltaflexivirus 2, pleurotus ostreatus alpha-like virus, and pleurotus ostreatus ambivirus 2, and three new viruses tentatively named pleurotus ostreatus deltaflexivirus 3, pleurotus ostreatus ambivirus 3 and pleurotus ostreatus lentinuvirus 1. The sequence characterization, genomic structure and phylogenetic relationships of these new viruses with other known viruses were also characterized. Further virus distribution patterns were explored with RT-PCR analysis of different strains isolated from different locations.

**Conclusions:**

In this study, a mixed infection with seven RNA viruses from a *P. ostreatus* strain was identified via deep sequencing, and their genomes were characterized. These findings advance our understanding of the diversity of viruses infecting *P. ostreatus* and provide critical insights for further research on virus‒host interactions and the development of viral elimination strategies.

**Supplementary Information:**

The online version contains supplementary material available at https://doi.org/10.1186/s12864-026-13166-z.

## Introduction

Of the commercially cultivated fungi, *Pleurotus ostreatus* (commonly known as the oyster mushroom) holds a prominent global position. It is a highly popular and economically viable species, with significant consumption documented in Southeast Asia, India, Europe, and Africa [1]. *P*. *ostreatus* is recognized as a nutritional powerhouse, rich in proteins, vitamins, minerals, and all nine essential amino acids required by the human body, underscoring its importance in the nutritional landscape [2]. Its cultivation is relatively straightforward, as it can be effectively grown on a variety of agricultural and forestry residues, including grain straw, corn cobs, cottonseed hulls, and bagasse [3]. The species exhibits a rapid growth rate and short cultivation cycle, making it particularly suitable for large-scale production.

Similar to other fungi, *P. ostreatus* is susceptible to viral infections. The first isolation of spherical viruses from malformed sporophores was reported in 1992 [4] with subsequent observations suggesting that reduced mycelial growth might be associated with the presence of double-stranded RNA (dsRNA) virus [5]. Since then, viruses have been successively identified in various *P. ostreatus* strains, including PO-Die-back [6], ASI2223, ASI2228 and Suhan [7], PO-South-Eastern Korea [8], ASI2596 [9], ASI2504 [10], Suhan-Southern Korea [11], Shin-Nong [12], TD300 [13], ASI2792 [14] and ZP6 [15]. Among these mycoviruses, oyster mushroom spherical virus (currently unclassified within *Tymovirales*) from PO-Die-back strain and pleurotus ostreatus deltaflexivirus 1 from ZP6 strain possess a positive single stranded RNA genome [8, 15]. In contrast, pleurotus ostreatus virus 1 (*Betapartitivirus*) from ASI2596, unassigned oyster mushroom isometric virus I and II, pleurotus ostreatus virus Shin-Nong and pleurotus ostreatus spherical virus have dsRNA genomes. The oyster mushroom spherical virus, identified as the causative agent of epidemic die-back disease, has been detected in Korea and China. It can be horizontally transmitted via hyphal anastomosis from infected to cured strains, vertically transmitted to progeny via basidiospores [16]. Infection with the oyster mushroom isometric virus has been associated with abnormal phenotypic characteristics, such as retarded mycelial growth, undeveloped stipes, and deformed caps on fruiting bodies [8]. In the TD300 strain, a mixed infection involving pleurotus ostreatus spherical virus and other mycoviruses led to strain degeneration [13], while the PoV-ASI2792 strain was found to inhibit growth rate and reduce mycelial dry weight [14]. In contrast, infected with pleurotus ostreatus virus 1 did not result in any discernible morphological or growth phenotypes [9].

The advent of high-throughput sequencing (HTS) has revolutionized the field of virus discovery, enabling the *de novo* identification of novel viruses without prior sequence information. This technology has significantly advanced our understanding of viral diversity and evolution, leading to the discovery of numerous previously unknown mycoviruses [17, 18]. Macrofungi have become a major focus for viral surveillance, with many mycoviruses identified in commercially cultivated species. For instance, 18 unique RNA viruses have been reported in *Agaricus bisporus* [19], 27 new viruses in *Lentinula edodes* [20–24], and 3 in *Auricularia heimuer* [25–27].

As a key component of Beijing’s food supply chain, the edible mushroom industry produced 66,000 metric tons with a production value of 370 million RMB in 2023. *P. ostreatus* is the predominant cultivated species in the city, accounting for over 50% of the total mushroom production. However, like other edible fungi, *P. ostreatus* cultivation in Beijing is also affected by mycoviral infections. In a previous study, we identified eight RNA mycoviruses coinfecting a *P. ostreatus* strain from Fangshan, seven of which were novel [28]. During a recent survey of a *P. ostreatus* production facility in Tongzhou, symptoms of weakened growth, malformed fruiting bodies, and reduced quality and yield were observed. To characterize the associated virome, the affected strain was isolated, purified and subjected to ribosomal RNA-depleted RNA sequencing. This analysis led to the identification and characterization of four previously reported and three novel RNA viruses and the virus distribution pattern was further investigated. The findings of this study contribute to a deeper understanding of the viral pathogens affecting *P. ostreatus* and provide valuable insights for future research on cultivation management and host-pathogen interactions.

## Materials and methods

### Sample collection and total RNA extraction

**Fig. 1:**
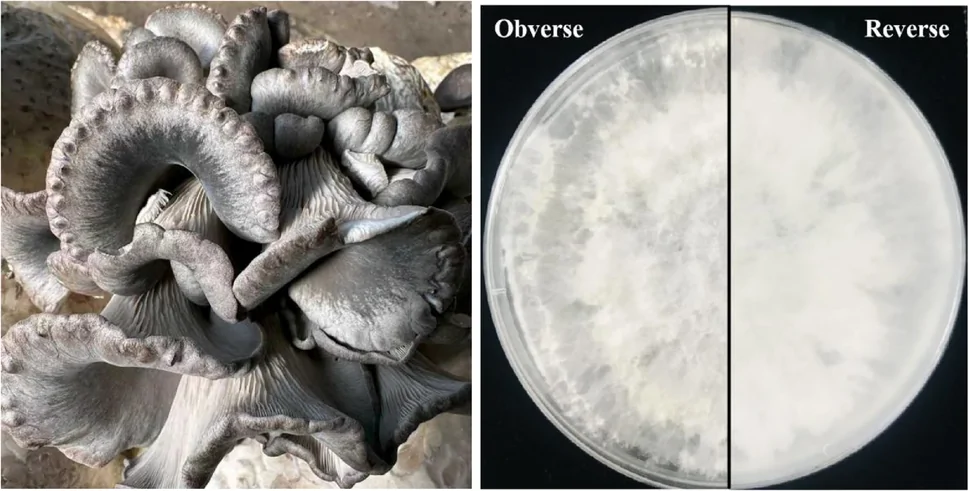
The symptom of malformed *Pleurotus ostreatus* fruiting bodies (left) and the colony morphology of the isolated strain cultured on PDA for 14 days at 25 °C (right)

A single malformed fruiting body of *P. ostreatus* was collected from Sanbaoxiang Agricultural Technology Park in Tongzhou District, Beijing and the mycelium was isolated from the fruiting body and cultured on potato dextrose agar plates at 25 °C for 14 days in the dark (Fig. 1). Approximately 0.5 g (wet weight) of mycelia were harvested, ground to a fine powder in liquid nitrogen using a mortar and pestle, and subjected to total RNA extraction using the RNAprep Pure Plant Plus Kit (polysaccharides & polyphenolics-rich) (Tiangen, Beijing, China) according to the manufacturer′s instructions. RNA quality and integrity were assessed using a Nanodrop spectrophotometer and an Agilent 2100/GX Bioanalyzer.

### High-throughput sequencing and bioinformatic analysis

Ribosomal RNA was removed from the total RNA using the Ribo-ZeroTM rRNA Removal Kit (Epicentre, Madison, WI, USA). A cDNA library was then constructed with the TruSeq RNA Sample Prep Kit v2 (Illumina, CA, USA) following the manufacturer′s protocol. The library was sequenced on an Illumina HiSeq 2500 platform (150 nt paired-end sequencing) at Youji Technology Co. Ltd. (Beijing, China). Raw sequencing data quality was evaluated using FastQC (version 0.12.1) and reads were trimmed to remove short reads, poly-A tails, poly-G regions, and adapter sequences using cutadapt (version 4.8). Clean reads were *de novo* assembled into contigs with Trinity v2.14.0. Virus-derived sequences were identified by Basic Local Alignment Search Tool (BLAST) searches against viral reference sequences from the National Centre for Biotechnology Information database.

### Virus detection and complete genome sequence determination

To validate candidate viruses identified from the sequencing data, sequence-specific primer pairs were designed using Primer Premier 5.0 based on the assembled contigs (Table S1). Total RNA was heat-denatured at 70 °C for 5 min, cooled on ice for 1 min, and reverse-transcribed using the PrimeScript II 1st Strand cDNA Synthesis Kit (Takara, Dalian, China). PCR amplification was performed in a 25 µL reaction mixture containing 1 µL cDNA, 1× LA *Taq* buffer, 10 mM dNTPs, 1 U LA *Taq* polymerase, 100 mM of each primer, and sterile double-distilled water. The thermal cycling conditions were as follows: initial denaturation at 94 °C for 5 min; 28 cycles of 94 °C for 30 s, 52 °C for 30 s and 72 °C for 1 min; and a final extension at 72 °C for 10 min [28]. To obtain complete genome sequences, overlapping primer pairs were designed for RT-PCR amplification as described above. The amplification products were purified, cloned into the pMD19-T vector, and transformed into *E. coli* DH5α competent cells. At least three independent clones for each fragment were sequenced. The 5′ and 3′ terminal sequences were determined using the SMARTer RACE 5′/3′ kit (TaKaRa, Dalian, China) following the manufacturer′s instructions (primers listed in Table S1).

### Sequence characterization and phylogenetic analysis

Potential open reading frames (ORFs) in the viral genomic RNA were predicted using the ORF Finder tool at NCBI (https://www.ncbi.nlm.nih.gov/orffinder/). Conserved and functional protein domains were identified through the Conserved Domain Database (www.ncbi.nlm.nih.gov/Structure/cdd/wrpsb.cgi/). Sequence similarity searches were conducted using NCBI BLASTx and BLASTp programs. For phylogenetic analysis, related viral genome sequences were retrieved from GenBank. Multiple sequence alignments were performed using Clustal W, and phylogenetic trees were constructed using the maximum-likelihood (ML) method implemented in the MEGA6.06 software, with the best-fit substitution model determined by model testing within the software. Branch support was evaluated with 1000 bootstrap replicates.

### Field survey of the identified viruses

To investigate the field prevalence of the candidate viruses, 46 *P. ostreatus* strains (10 from Shunyi, 10 from Tongzhou, 12 from Miyun, and 14 from Fangshan) were isolated from mushroom spawns obtained from the production farms. Mycelia were cultured on potato dextrose agar plates at 25 °C for 10 days. Total RNA was extracted from the mycelia, and RT-PCR was performed as described above (primers listed in Table S1). The resulting amplicon products were analyzed by electrophoresis on 1.0% agarose gels, stained with ethidium bromide, and visualized under UV light.

## Results

### Deep sequencing identification of candidate mycoviruses

After adaptor trimming and low-quality read removal, a total of 118,973,697 clean reads were obtained and assembled using Trinity into 53,864 contigs. BLASTx analysis against the non-redundant protein database revealed that the majority of contigs mapped to the host *P. ostreatu*s genome, while a subset showed significant amino acid sequence identity to known mycoviruses. Six candidate RNA viruses were identified: oyster mushroom spherical virus, pleurotus ostreatus deltaflexivirus 1, Botrytis cinerea alpha-like virus 1, Heterobasidion ambi-like virus 6, Heterobasidion ambi-like virus 15, and Armillaria bunya-like virus 2 (Table 1). Most candidate viruses shared less than 80% amino acid sequence identity with known viral species, suggesting they might represent novel isolates. The oyster mushroom spherical virus was an exception, showing high sequence similarity to existing isolates. The presence of all the candidate viruses was confirmed by RT‒PCR using sequence specific primers (Table S1, Figure S1), followed by Sanger sequencing of the amplification products.

**Table 1:** Candidate mycoviruses identified in the sequenced sample

BLASTx results*	Reads no.	Virus taxon	Contig length	aa sequence identity (%)
Oyster mushroom spherical virus	10,234	unclassified/+ssRNA	5745 nt	99.7
Pleurotus ostreatus deltaflexivirus 1	19,846	*Deltaflexiviridae/*+ssRNA	7733–7746 nt	73.1–76.0
Botrytis cinerea alpha-like virus 1	10,312	*Togaviridae/*+ssRNA	8146 nt	35.3
Heterobasidion ambi-like virus 6	8,964	unclassified/circular RNA	11,930 nt	37.3
Heterobasidion ambi-like virus 15	8,631	unclassified/circular RNA	8060 nt	37.6
Armillaria bunya-like virus 2 RNA1 RNA2	10,035 12,411	unclassified*/-*ssRNA	7099 nt 2470 nt	31.4 34.2

*These viruses were initially identified by BLASTx with the assembled contigs, and the final names of the viruses corresponding to oyster mushroom spherical virus, pleurotus ostreatus deltaflexivirus 1, botrytis cinerea alpha-like virus 1, Heterobasidion ambi-like virus 6, Heterobasidion ambi-like virus 15 and Armillaria bunya-like virus 2 were proposed as oyster mushroom spherical virus, pleurotus ostreatus deltaflexivirus 2/pleurotus ostreatus deltaflexivirus 3 (two deltaflexiviruses), pleurotus ostreatus alpha-like virus, pleurotus ostreatus ambivirus 2, pleurotus ostreatus ambivirus 3 and pleurotus ostreatus lentinuvirus 1 respectively

### Complete genome sequencing and characterization of candidate viruses

To comprehensively characterize the candidate viruses, near-complete or complete genome sequences were obtained through RT‒PCR coupled with rapid amplification of cDNA ends (RACE) for terminal genome regions (Figure S2). Genome architecture, sequence features, and phylogenetic relationships with other reported viruses were systematically analyzed.

### Oyster mushroom spherical virus and pleurotus ostreatus alpha-like virus

**Fig. 2:**
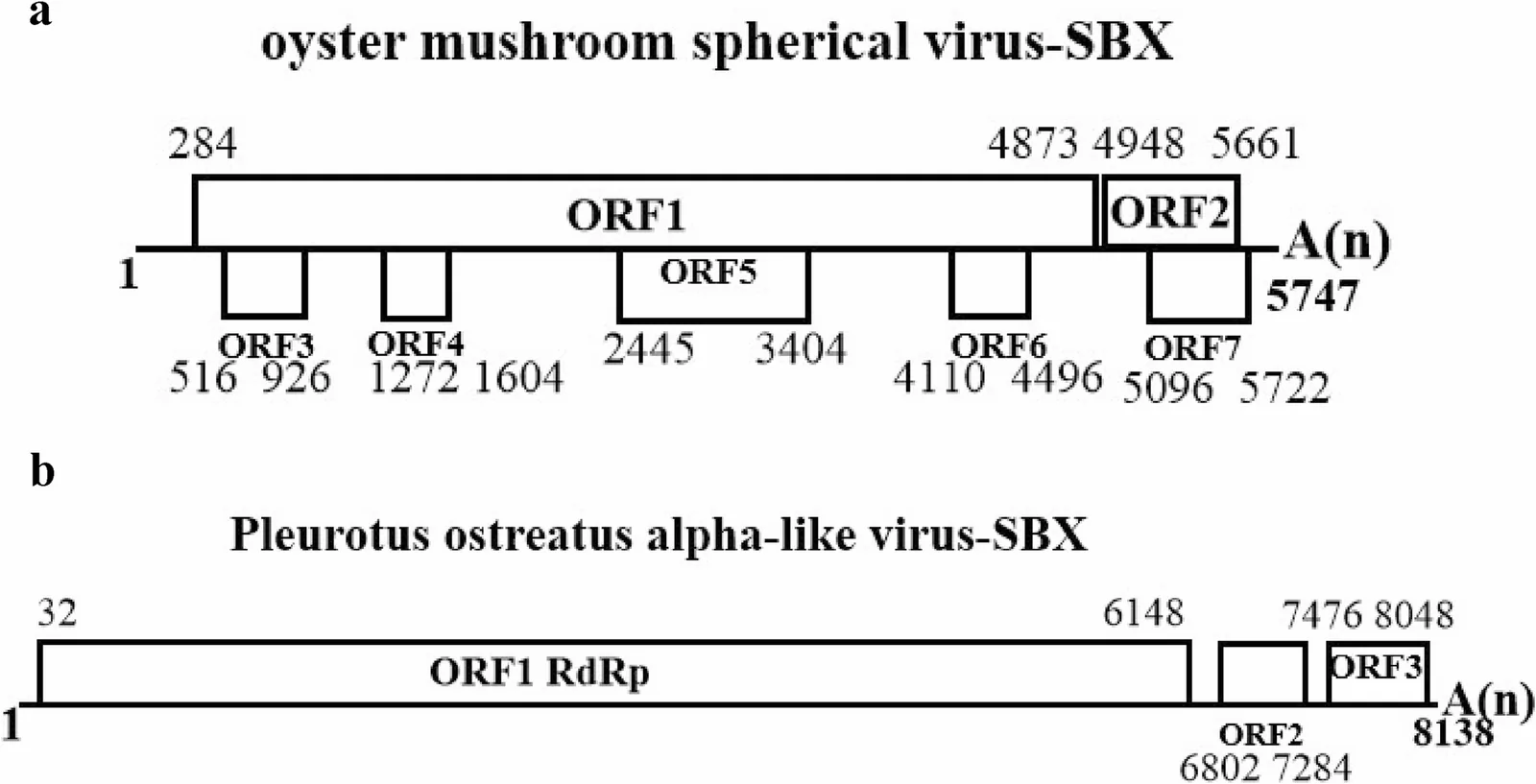
Genome structure of oyster mushroom oyster mushroom spherical virus-SBX (**a**) and pleurotus ostreatus alpha-like virus-SBX (**b**). Open reading frames (ORFs) are represented by boxes, and each position in the genome is also indicated

The complete genome of oyster mushroom spherical virus-SBX (PV524678) was 5747 nt in length, contained seven ORFs (Fig. 2a). It shared 99.5% nt sequence identity with the Chinese isolates (OL546221 and PQ665306) and 79.1% identity with a South Korea isolate (AY182001). ORF1 (nt 284–4873) encoded an RNA-dependent RNA polymerase (RdRp) of approximately 168.9 kDa, featuring conserved helicase domains with GTAGCGKS and GDD motifs. ORF2 (nt 4948–5661) encoded a 24.0 kDa capsid protein without identifiable motifs. ORF3-ORF6, embedded within ORF1, encoded putative polyproteins of 14.5, 11.6, 34.5, and 13.3 kDa, respectively, with unknown functions. ORF7 (nt 5096–5722) overlapped with ORF2, encoding a 22.0 kDa protein. Pleurotus ostreatus alpha-like virus-SBX (PV524679) was 8138 nt in length with three putative ORFs (Fig. 2b), sharing 98.7% nt sequence identity with the previously reported isolate BJ-12 (PP848159).

### Genome characterization of two deltaflexiviruses

**Fig. 3:**
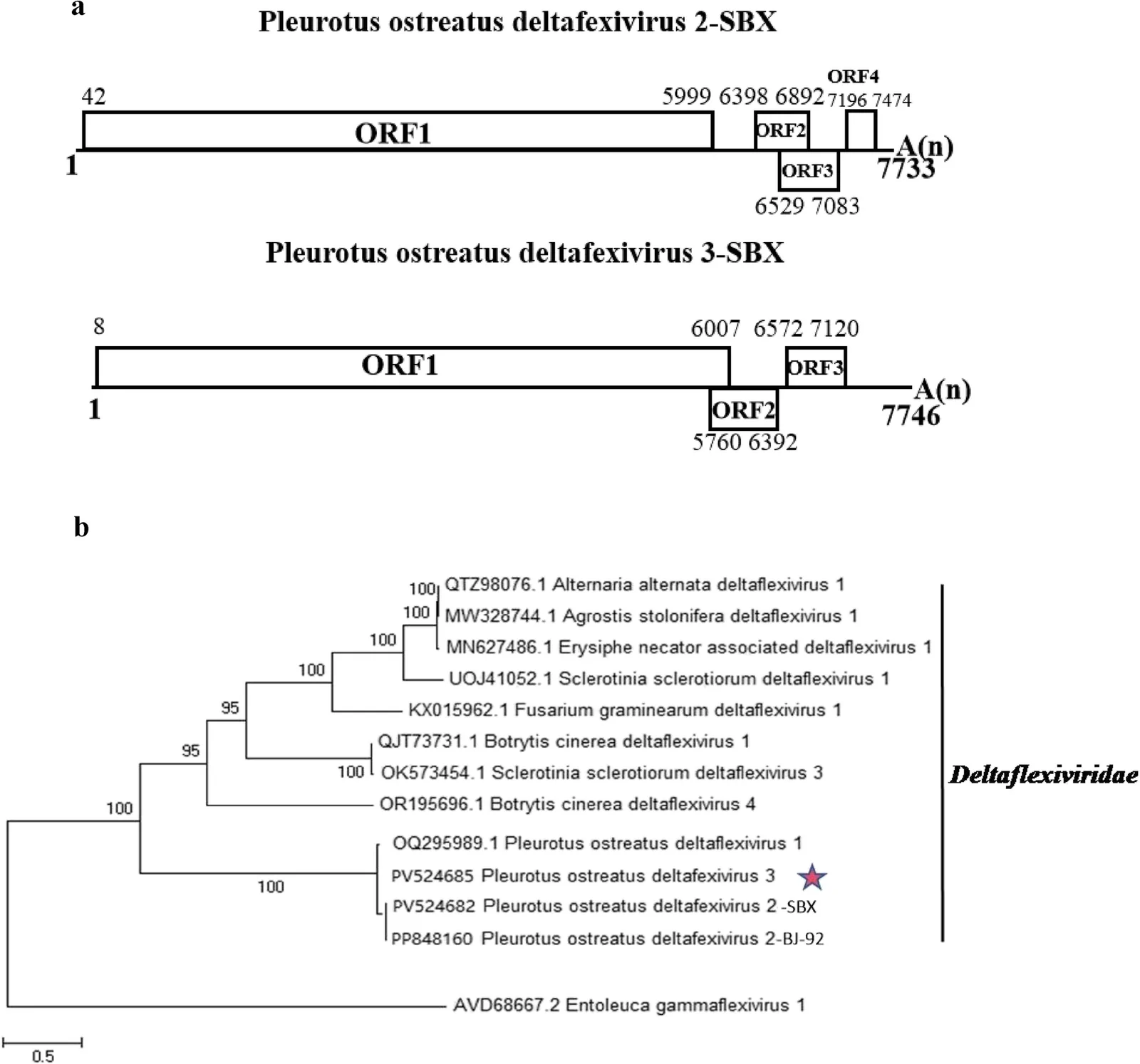
Genome structure and phylogenetic analyses of pleurotus ostreatus deltaflexiviruses. **a** Genome organization of Pleurotus ostreatus deltaflexivirus 2 and Pleurotus ostreatus deltaflexivirus 3. Open reading frames (ORFs) are represented by boxes, and each position in the genome is also indicated. **b** Phylogenetic tree constructed via the ML method on the basis of the aa sequences of REP different deltaflexiviruses, with Entoleuca gammaflexivirus as an outgroup

The complete genomes of the two deltaflexiviruses were successfully cloned and designated as pleurotus ostreatus deltaflexivirus 2-SBX (PV524682) and pleurotus ostreatus deltaflexivirus 3-SBX (PV524685). The 7733 nt genome of pleurotus ostreatus deltaflexivirus 2-SBX encoded four putative proteins (Fig. 3a) and shared 99.1% nt sequence identity with the isolate BJ-92 (PP848160). Pleurotus ostreatus deltaflexivirus 3-SBX (7746 nt) contained three ORFs (Fig. 3a), distinguishing it from pleurotus ostreatus deltaflexivirus 1 and pleurotus ostreatus deltaflexivirus 2. The largest ORF, ORF1 (nt 8-6007), encoded a 221.34 kDa polyprotein (1999 aa) containing three conserved domains: viral methyltransferase (pfam01660), viral helicase 1 (pfam01443), and RNA-dependent RNA polymerase (RdRP_2, pfam00978). BLAST analysis revealed 76.01% aa identity with the ORF1 polyprotein of pleurotus ostreatus deltaflexivirus 1. ORF2 (nt 5760–6392) overlapped with ORF1, encoding a 23.08 kDa protein (210aa), while ORF3 (nt 6572–7120) encoded a 182-aa protein showing 72.25% identity with its counterpart in pleurotus ostreatus deltaflexivirus 1.

Phylogenetic analysis based on replication-associated protein (REP) sequences placed pleurotus ostreatus deltaflexivirus 3-SBX within the *Deltaflexivirus* genus, clustering closely with pleurotus ostreatus deltaflexivirus 1 and pleurotus ostreatus deltaflexivirus 2 (Fig. 3b). Species demarcation criteria in this genus typically include host range and genome organization, but sequence-based thresholds remain undefined. As an initial approach, the criteria established for the related *Alpha-* and *Betaflexiviridae* families, which currently use thresholds of < 72% nt identity or < 80% aa identity in the REP, were considered. The REP of pleurotus ostreatus deltaflexivirus 3-SBX shared < 70% nt and < 79% aa sequence identity with those of pleurotus ostreatus deltaflexivirus 1 and pleurotus ostreatus deltaflexivirus 2, falling below the proposed thresholds for establishing new species. These findings suggested that pleurotus ostreatus deltaflexivirus 3-SBX represented a distinct species within the *Deltaflexivirus* genus.

### Characterization of identified ambiviruses

**Fig. 4:**
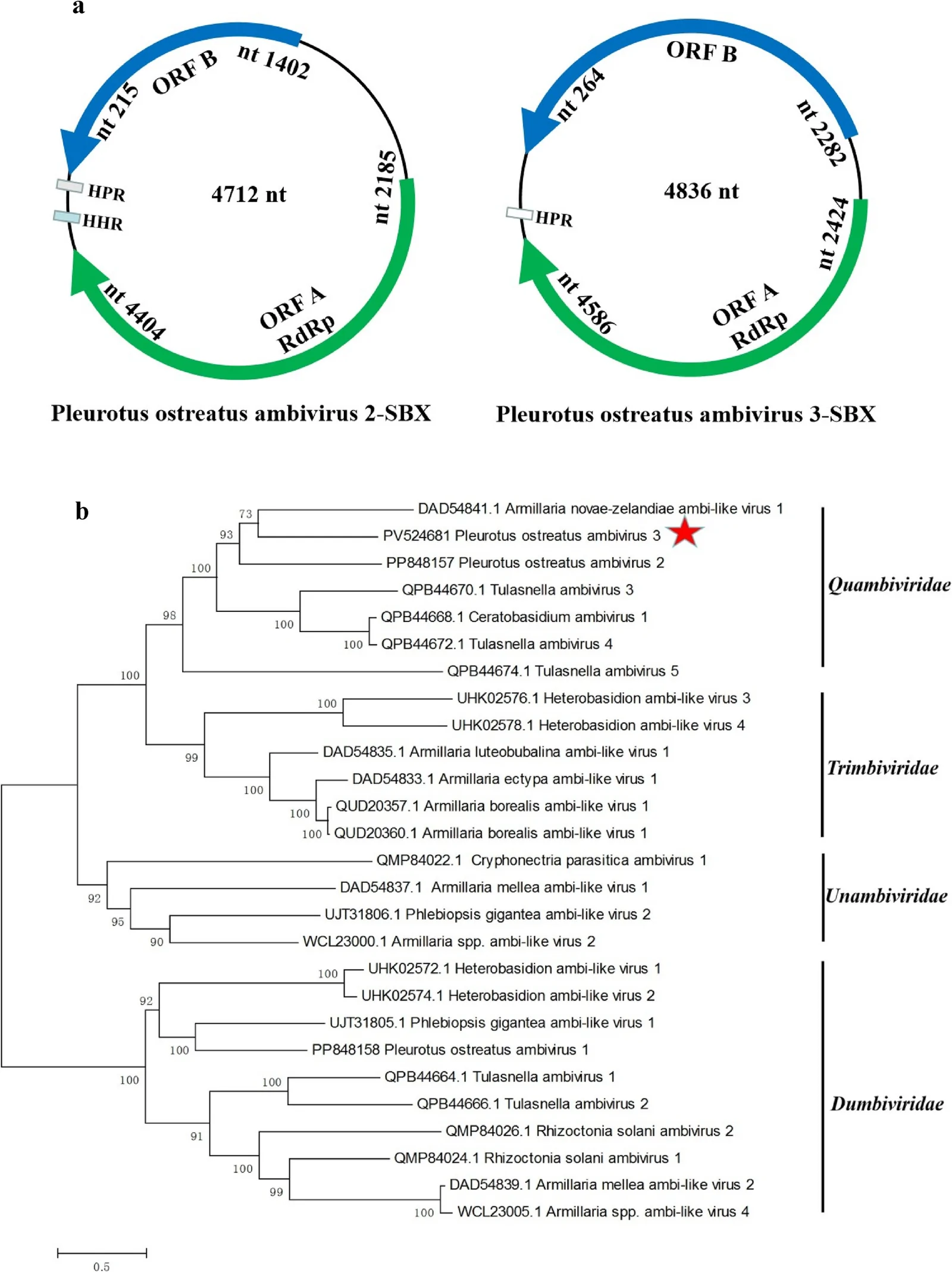
Genome structure and phylogenetic analyses of pleurotus ostreatus ambiviruses. **a** Genome organization of pleurotus ostreatus ambivirus 2 and pleurotus ostreatus ambivirus 3. Open reading frames (ORFs) are represented by different arrows, and each position in the genome is indicated. The ribozyme types present are also indicated. **b** Phylogenetic tree constructed via the ML method on the basis of the aa sequences of RdRp of different ambiviruses

Two ambiviruses were identified: pleurotus ostreatus ambivirus 2-SBX (PV524680) and a tentatively named pleurotus ostreatus ambivirus 3-SBX (PV524681) (Fig. 4a). Pleurotus ostreatus ambivirus 2-SBX (4712 nt) shared 94.2% nt sequence identity with the reported isolate BJ-61(PP848157**)**. The circular genome of pleurotus ostreatus ambivirus 3-SBX (4836 nt) contained two non-overlapping ORFs in ambisense orientation (Fig. 4a). ORF A was located in the sense orientation (nt 2424–4586) and encoded a 720 aa putative protein with an estimated molecular mass of 82.95 kDa. BLAST analysis revealed that it shared 26.1–48.4% aa sequence identity with the RdRp of various ambi-like viruses, including Trametes versicolor ambi-like virus 1, Heterobasidion ambi-like virus 6, Amanita strobiliformis ambi-like virus 1, and Phlebiopsis gigantea ambi-like virus 2. No conserved domains were identified, but the reported typical motifs A ((T/L/I)YD(Y/L)(S/T)SFT(S/T)(S/E/N)(L/M/H)), B (LG(A/V/I)(Q/Y)GNI(G/A/S/N)(F/L/S)(S/C)T(L/A/M)LHG), and C ((V/C)(V/A/I)GDD(A/G)) in the palm subdomain of ambivirus RdRp were present. The antisense strand encoding ORF B (nt 2282–264) was estimated to be 672 aa long with an estimated molecular mass of 73.77 kDa and shared 22.35–40.31% aa sequence identity with the corresponding proteins of other ambiviruses. No functional motifs were present in this protein. Ribozymes were reported to be located at the C-termini of both ambivirus ORFs within the non-protein-coding region of the genome. Using the untranslated regions of pleurotus ostreatus ambivirus 2-SBX and pleurotus ostreatus ambivirus 3-SBX as inputs in the Rfam database (https://rfam.org/search), hammerhead (HHR) and hairpin (HPR) ribozyme types were identified in pleurotus ostreatus ambivirus 2-SBX, whereas only HPR was present in pleurotus ostreatus ambivirus 3-SBX (Fig. 4a). To further clarify the taxonomic status of pleurotus ostreatus ambivirus 3*-*SBX and elucidate its relationship with other reported ambiviruses, an ML phylogenetic tree was constructed on the basis of the RdRp aa sequences. Pleurotus ostreatus ambivirus 3-SBX clustered into the *Quambiviridae* clade with pleurotus ostreatus ambivirus 2 (Fig. 4b).

### Pleurotus ostreatus lentinuvirus 1-a novel species in *Lentinuvirus*

**Fig. 5:**
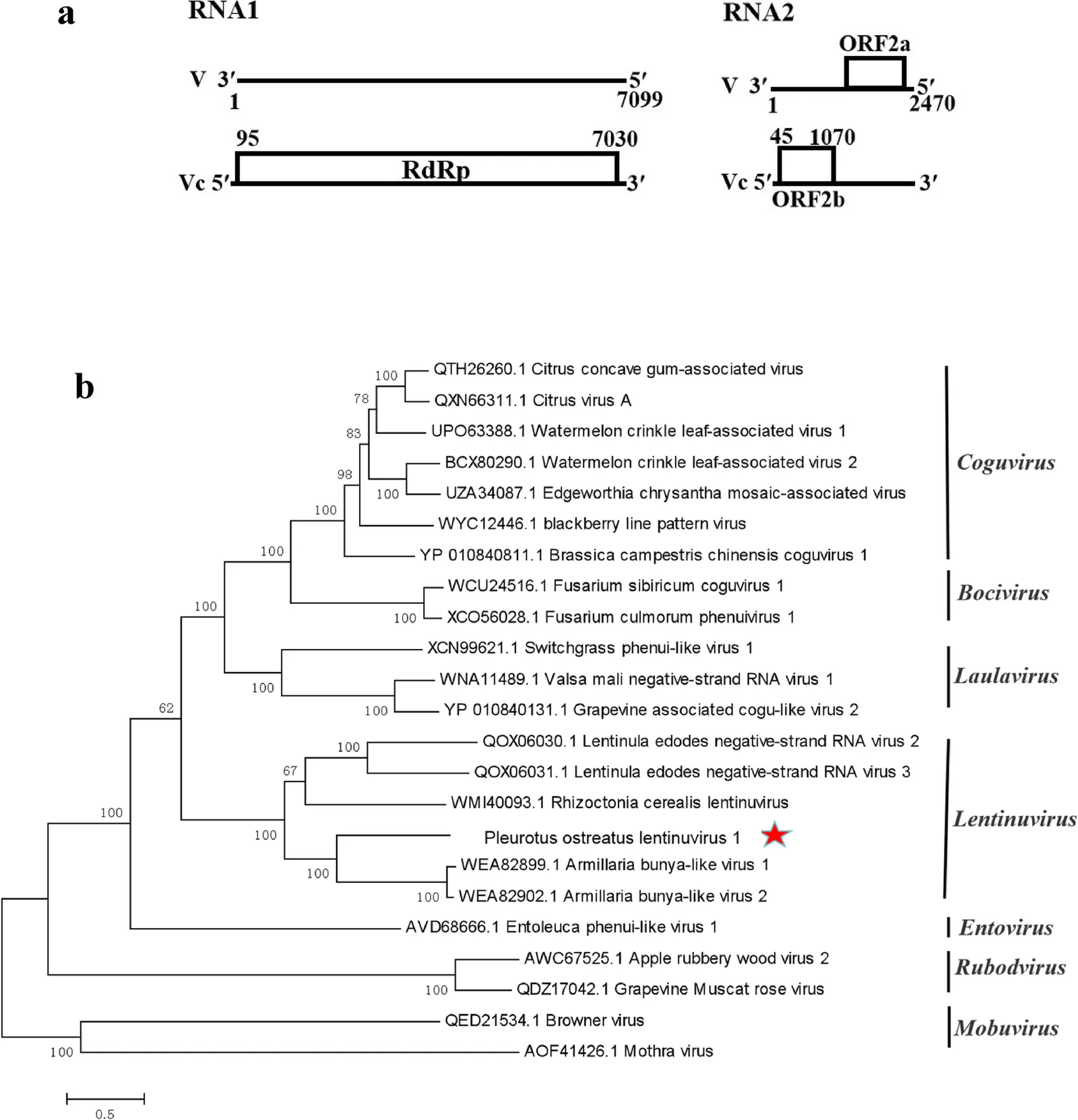
Genome structure and phylogenetic analyses of pleurotus ostreatus lentinuvirus 1 (PoLV1). **a**: Genome organization of PoLV1. Open reading frames (ORFs) are represented by boxes, and each position in the genome is also indicated. **b**: Phylogenetic tree constructed via the ML method on the basis of the amino acid sequences of the RdRp genes of different viruses in *Phenuiviridae*

The complete genome of pleurotus ostreatus lentinuvirus 1-SBX consisted of two single-stranded segments: RNA1 (7099 nt, PV524683) and RNA2 (2470 nt, PV524684) (Fig. 5a). RNA1 encoded a 2311 aa protein of 266.18 kDa with a conserved Bunya_RdRp domain (pfam04196, 7.63e-33, aa 865–1298), indicating that this protein may be involved in virus replication. BLASTp analysis revealed that it shared 40.12%, 39.87%, 35.57% and 32.15% aa sequence identity with the L protein of Armillaria bunya-like virus 2, Armillaria bunya-like virus 1, Rhizoctonia cerealis lentinuvirus and Lentinula edodes negative-strand RNA virus 2, respectively. RNA2 was ambisense and was likely transcribed by RdRp to form a subgenomic virus-complementary mRNA that encoded the ORF2b protein from a viral antigenome RNA2, which shared 34.78% aa sequence identity with the nucleocapsid-like protein of Armillaria bunya-like virus 1. RNA2 was also transcribed to form a subgenomic virus-sense mRNA encoding the ORF2a protein (359 aa of 40.75 kDa) from the viral genome RNA2, which had 28.76% aa sequence identity (63% coverage) with the putative movement protein of Armillaria bunya-like virus 1. The two ORFs were separated by a noncoding 306-nt intergenic region that also formed a long A/U rich stem-loop structure as previous reported (Figure S3). For many segmented (−)ssRNA viruses, the RNA segments shared the first 10 nucleotides at 3′ terminus (3′-ACACAAAGAC …) and the first nine nucleotide at 5′ terminus (… UCUUUGUGU-5′). Moreover, the first 9 nucleotide sequences of 3′ and 5′ termini of each RNA strand were complementary to each other [20]. In pleurotus ostreatus lentinuvirus 1, however, the conserved sequence ACACAAAGA was present only in RNA2, and the 5′ terminal sequence (… UCUUUGUGU-5′) was absent from both RNA segments. An ML phylogenetic tree was constructed using RdRp protein sequences derived from representative members of the family *Phenuiviridae*. Pleurotus ostreatus lentinuvirus 1 clustered together with Armillaria bunya-like virus 2, Armillaria bunya-like virus 1, Rhizoctonia cerealis lentinuvirus and Lentinula edodes negative-strand RNA virus 2 (Fig. 5b). Genus demarcation in *Phenuiviridae* is based on virus phylogenetic relationships, genome organization and ecology, and viruses assigned to different species have less than 95% identity in the amino acid sequence of the RdRp. Pleurotus ostreatus lentinuvirus 1-SBX had a similar genome structure, clustered together with Lentinula edodes negative-strand RNA virus 2, the typical member in the genus *Lentinuvirus*, and shared 32.15% aa sequence identity with Lentinula edodes negative-strand RNA virus 2 RdRp. These findings indicated that pleurotus ostreatus lentinuvirus 1-SBX was a new species in *Lentinuvirus*.

### Field distribution pattern of candidate viruses

To explore the prevalence of the seven identified viruses in different locations, field samples were collected and RT-PCR was performed with a specific primer pair for each virus (Table S1). A total of 46 samples from four regions revealed a high prevalence of mycoviruses, with an overall infection rate of 63.0% (29/46 samples) (Table 2). The overall infection profile was dominated by pleurotus ostreatus deltaflexivirus 2 (54.3%) and oyster mushroom spherical virus (50.0%), suggesting these were endemic and widespread in the sampled populations (Table 2). The Tongzhou region exhibited the highest infection rate (70.0%) and the highest incidence of pleurotus ostreatus deltaflexivirus 2 (70.0%). Notably, Shunyi showed a distinct profile with markedly high prevalence of pleurotus ostreatus ambivirus 2 (60.0%), a virus that remained below 30% incidence in all other regions (Table 2). Pleurotus ostreatus lentinuvirus 1 demonstrated a restricted distribution, being detected only in Tongzhou (30%), and completely absent from the other samples. This may indicate a geographically limited spread or specific host-strain interaction. Fangshan displayed the most diverse viral community, with no single virus dominating (Figure S4). Analysis of viral co-infection patterns across four sampled regions revealed a striking absence of single infections and pronounced geographic heterogeneity in complexity (Figure S4). While co-infection with 2–7 viruses was ubiquitous, the distribution varied significantly: Tongzhou exhibited the most complex profiles, including the only instances of 7-virus co-infection and the highest frequency of 4- and 5-virus combinations. In contrast, Miyun displayed the simplest patterns, dominated by 2-virus co-infections with no high-order (> 5 virus) cases. Shunyi and Fangshan showed intermediate profiles. This structured variation suggests that local ecological or agronomic factors strongly influence viral community assembly and host susceptibility.

**Table 2:** Field survey of different viruses detected by RT-PCR

Location	No. of samples(total/virus infected)	Percentage of tested samples with virus						
		Oyster mushroom spherical virus	Pleurotus ostreatus deltaflexivirus 2	pleurotus ostreatus alpha-like virus	pleurotus ostreatus ambivirus 2	Pleurotus ostreatus deltaflexivirus 3	Pleurotus ostreatus ambivirus 3	Pleurotus ostreatus lentinuvirus 1
Shunyi	10/6	50% (5/10)	40% (4/10)	20% (2/10)	60%(6/10)	20%(2/10)	30%(3/10)	0%(0/10)
Tongzhou	10/7	60% (6/10)	70% (7/10)	40% (4/10)	30%(3/10)	20%(2/10)	50%(5/10)	30%(3/10)
Miyun	12/6	33% (4/12)	33% (4/12)	25% (3/12)	50%(6/12)	33%(4/12)	17%(2/12)	0%(0/12)
Fangshan	14/10	57% (8/14)	71% (10/14)	36% (5/14)	29%(4/14)	21%(3/14)	29%(4/14)	0%(0/14)
Total	46/29	50%(23/46)	54%(25/46)	30%(14/46)	41%(19/46)	24%(11/46)	30%(14/46)	7%(3/46)

## Discussion

High-throughput sequencing (HTS) has been widely used for mycovirus exploration, leading to the identification of numerous previously unreported mycoviruses. Most of these have been found in mixed infections; for example, up to 17 mycoviruses have been characterized in the avirulent *R*. *solani* isolating DC17 and 16 novel RNA mycoviruses coinfecting the *Rhizoctonia zeae* strain D40 [29, 30]. Similarly, the mycovirome of edible fungi has also been investigated, and many new viruses have been identified [19–28]. In our previous study, a set of novel mycoviruses from a single *P. ostreatus* strain using a similar HTS approach was reported [28], which provided a glimpse into the viral diversity within this mushroom species, but it remained unclear whether other strains harbor similar or distinct viral communities. Here, we characterized the virome of a second, independently sourced *P. ostreatus* strain and simultaneously identified seven RNA mycoviruses, comprising four previously reported viruses (oyster mushroom spherical virus, pleurotus ostreatus deltaflexivirus 2, pleurotus ostreatus alpha-like virus, and pleurotus ostreatus ambivirus 2) and three novel viruses tentatively designated as pleurotus ostreatus deltaflexivirus 3, pleurotus ostreatus ambivirus 3 and pleurotus ostreatus lentinuvirus 1. These findings provided critical evidence for strain-dependent virome composition in *P. ostreatus* and expanded the known mycovirus repertoire. Further broader investigation of viral diversity in *P. ostreatus* is therefore needed, ideally by screening a larger collection of strains from diverse geographic and cultivation backgrounds.

With the application of deep sequencing, the number of mycoviruses with +ssRNA genomes has been steadily increasing, constituting the majority of known mycoviruses [31]. To date, four +ssRNA mycoviruses, oyster mushroom spherical virus, pleurotus ostreatus alpha-like virus, pleurotus ostreatus deltaflexivirus 1, and pleurotus ostreatus deltaflexivirus 2, have been reported in *P. ostreatus.* These four viruses were detected together with a new deltaflexivirus, pleurotus ostreatus deltaflexivirus 3. Unlike pleurotus ostreatus deltaflexivirus 1 and pleurotus ostreatus deltaflexivirus 2, which contained a small ORF (ORF4), pleurotus ostreatus deltaflexivirus 3 lacked this ORF and instead possessed three ORFs as predicted by ORF Finder. Members in the family *Deltaflexiviridae* were once thought to be capsidless because no virus particles have yet been found until recently the newly discovered Fusarium oxysporum icosahedral virus 1 which harbored a small ORF4 encoding a single jelly-roll -like coat protein (SJR-CP) that constituted the icosahedral virions [32]. Further sequence analysis and structure prediction indicated that most of the deltaflexiviruses including pleurotus ostreatus deltaflexivirus 1 have this similar icosahedral virions composed of SJR-CPs. The potential ORF4 encoded polypeptide in pleurotus ostreatus deltaflexivirus 2 was 92 aa long, shorter than that in pleurotus ostreatus deltaflexivirus 1 and no SJR-like structure was identified with using HHpred or pLM-BLAST searches. In pleurotus ostreatus deltaflexivirus 3, ORF4 was absent. Using the ORF3 polypeptides of pleurotus ostreatus deltaflexivirus 2 (184 aa) and pleurotus ostreatus deltaflexivirus 3 (182 aa) as queries in the pLM-BLAST search (https://toolkit.tuebingen.mpg.de/tools/plmblast), we identified 61 and 58 sequences, respectively, that shared structural homology with the SJR-CP (capsid protein) fold. No such homology was found for the ORF3 protein of pleurotus ostreatus deltaflexivirus 1. These in silico predictions raised the intriguing possibility that the ORF3 encoded polypeptides in pleurotus ostreatus deltaflexivirus 2 and pleurotus ostreatus deltaflexivirus 3 may form the icosahedral virions. This coding capacity may be involved in special host fitness and compatible host–virus interactions. However, this hypothesis required further experimental validation.

Since the –ssRNA mycoviruses related to the *Nyaviru*s within *Mononegavirales* were reported *from Erysiphe pisi* and *Sclerotinia homeocarpa*, diverse mycoviruses with −ssRNA genomes have been identified, including mymonaviruses (order *Mononegavirales*), phenui- and bunya-like viruses, and yueviruses and aspivirus-related viruses via the metagenomic approach [18]. The genomes of these –ssRNA viruses are monopartite, bi, tri- or multipartite. Monopartite LeNSRV1 and bipartite LeNSRV2 were the first two –ssRNA viruses from the edible fungus *Lentinula edodes* [20]. Similarly, pleurotus ostreatus mymonavirus 1, related to LeNSRV1, was previously reported from *Pleurotus ostreatus* [28], and here pleurotus ostreatus lentinuvirus 1, with a bipartite RNA genome related to LeNSRV2, was identified. Although conserved terminal sequences were only detected in the RNA2 3′ terminal, segment organization and intergenic feature of RNA2 and the phylogenetic analysis supported that pleurotus ostreatus lentinuvirus 1 was a new member in *Lentinuvirus.* These findings demonstrated that –ssRNA viruses may also be common in macrofungi, which enhanced our understanding of the diversity, evolution and spread of −ssRNA viruses in fungi.

Ambiviruses represent a recently characterized class of mobile genetic elements featuring circular RNA genomes. These genomes contain two ambisense-oriented ORFs, each terminating with hammerhead (HHR), hairpin (HPR), or other ribozyme motifs [33]. Initially, identified in Agaricomycete and Sordariomycete fungi, subsequent metatranscriptomic analyses revealed thousands of ambivirus-like sequences across diverse fungal taxa [34, 35]. Phylogenetic studies propose their classification into four distinct families: *Dumbiviridae*, *Quambiviridae*, *Trimbiviridae*, and *Unambiviridae*. In our investigation, we identified contigs exhibiting dimeric sequences. Following sequence amplification, we detected two ambiviruses coinfecting with other mycoviruses. Notably, bioinformatic analyses predicted the presence of HPR ribozyme structure in the novel ambivirus pleurotus ostreatus ambivirus 3. Viral elimination experiments in Armillaria borealis demonstrated no measurable impact on host growth kinetics or phenotypic characteristics [36]. However, the potential biological effects of pleurotus ostreatus ambiviruses remain to be elucidated through further investigation.

Coinfection by multiple mycoviruses in fungal strains is a well-documented phenomenon. While most mycoviruses establish asymptomatic infections in their hosts, certain strains exhibit either hypovirulent (magnaporthe oryzae chrysovirus 1 in *Chrysoviridae*, sclerotinia gemycircularvirus 1 in *Genomoviridae*) or hypervirulent characteristics (beauveria bassiana polymycovirus 1 in *Polymycoviridae*, alternaria alternata chrysovirus 1 in *Chrysoviridae*) [37]. Field survey revealed a high overall prevalence (63.0%) of mycoviruses in cultivated *Pleurotus ostreatus*, dominated by two widespread viruses (pleurotus ostreatus deltaflexivirus 2 and oyster mushroom spherical virus). Striking geographic heterogeneity was observed: the distribution of specific viruses like pleurotus ostreatus mymonavirus 1 (restricted to Tongzhou) and pleurotus ostreatus ambivirus 2 (highly prevalent in Shunyi) suggested strong influences from local ecological or agronomic factors. The complete absence of single-virus infections in all 46 field samples was a striking pattern with potential biological implications. The certain viruses in *P. ostreatus* may depend on co-infecting partners for stable maintenance, replication or transmission, analogous to helper-dependent virus systems, or the mixed infections might confer selective advantages (e.g., reduced fitness costs or enhanced transmission) under commercial cultivation conditions [38, 39]. However, methodological limitations cannot be ruled out: low-titer co-infections may fall below the detection threshold of RT-PCR, and our sampling from cultivated environments might not represent the full diversity of naturally occurring single infections. Distinguishing these possibilities will require experimental co-infection studies and deeper sequencing of field isolates. The complex interplay between mycoviruses and their fungal hosts is influenced by multiple factors, including growth media composition, temperature, the presence of other mycoviruses, and the host genotype [40]. No significant difference in growth rate was observed between the isolated virus-infected and virus-free strains on PDA plates. We tried to eliminate the viruses with high-temperature subculturing to gain single virus infected strains to explore the biological characterization of the identified viruses, but no such strains were gained, which constrained the interpretative scope of our findings. To definitively assess the phenotypic consequences of these mycoviral infections, systematic application of established curing techniques (e.g., single-basidiospore isolation or protoplast regeneration) is required and subsequent analyses should target critical physiological and agronomic traits in *P. ostreatus*, such as growth dynamics, morphology, sporulation efficiency, pigmentation, fruiting body productivity, and the biosynthesis of nutrients and bioactive metabolites.

## Supplementary Information

Supplementary Material 1.

Supplementary Material 2.

## Data Availability

All the genome sequences were submitted to the NCBI GenBank with the acc. no. PV524678-PV524685, and the corresponding virus name was detailed in the MS. The raw sequence data reported in this paper have been deposited in the Genome Sequence Archive (Genomics, Proteomics & Bioinformatics 2025) in National Genomics Data Center, China National Center for Bioinformation / Beijing Institute of Genomics, Chinese Academy of Sciences (GSA: CRA045034) that are publicly accessible at https://ngdc.cncb.ac.cn/gsa. Standardized binomial (Linnaean) format of the viruses were as follows: Deltaflexivirus duopleurotii-pleurotus ostreatus deltaflexivirus 2; Orthoquambivirus duopleurotii-pleurotus ostreatus ambivirus 2; Deltaflexivirus tripleurotii-pleurotus ostreatus deltaflexivirus 3; Orthoquambivirus tripleurotii-pleurotus ostreatus ambivirus 3; Lentinuvirus pleurotii-pleurotus ostreatus lentinuvirus 1; Deltaflexivirus unipleurotii -pleurotus ostreatus deltaflexivirus 1; Betapartitivirus unipleuroti-pleurotus ostreatus virus 1.
