# Supplementary material for: Deep sequencing of single Pleurotus ostreatus strain revealed mixed infection of seven RNA viruses
Source: BMC Genomics. 2026 Jul 17;27:843. doi: 10.1186/s12864-026-13166-z (PMC13660232; doi:10.1186/s12864-026-13166-z)
Supplement: Supplementary file 1 [file 12864_2026_13166_MOESM1_ESM.doc]

Supplemental Table

Table S1 Primer pairs used in this study

| Primer pairs used for detection and complete genome sequencing | | |
| --- | --- | --- |
| Virus name | Forward (5'-3') | Reverse (5'-3') |
| oyster mushroom spherical virus* | CTCAAAACAA CGTCGCCTTA ATGCCG | TTGTTAAGAT CGTTCTTCAG ATGCC |
| Pleurotus ostreatus deltaflexivirus 2* | CTGCCGGCGA TGTTCCCGAG CTTTAT | AGAGGGGAAC AGTAGACGTG CAAGC |
| Pleurotus ostreatus deltaflexivirus 3* | TGCGCCTGTT ACTGCTTCTG CTATTC | ACACCAGGAG CGAGCTCAAA GTAAG |
| Pleurotus ostreatus alpha-like virus* | GATGTTCGAC AAGGTTATAG ACCCG | ACCATTGAGG TGGTGGATGT AACCG |
| Pleurotus ostreatus ambivirus 2* | TGCGGAAGGT CGAGAGGAAT TTTGAT | GACGACCTCC CCGTCACGGA GAGGTT |
| Pleurotus ostreatus ambivirus 3 * | GCGGCTGTGG CAACCAAGTG TTTGC | CATATGTAAG GAGGAGTTCA TCCTC |
| Pleurotus ostreatus lentinuvirus 1* | CGCTTTCAGA TATCTCCGAT ACTAT | AATTTGTATA TGCTCAGACT AGGCC |
| Pleurotus ostreatus deltaflexivirus 2 S1 | CTTTGCCTCA CCGTTTTTGA TATCTAC | GAAGTGCTCC AAGATAGAAC CAGCCAG |
| Pleurotus ostreatus deltaflexivirus 2 S2 | CCTCTCCCAG CTAATGCAGG AGTTGGG | CCAGTCCACG GGAGAATTAG CCACAG |
| Pleurotus ostreatus deltaflexivirus 2 S3 | AATGGGACTT TGCATAAGGG ACTGTTG | ACC AGCTTCTGCA CTGAAGTACA ACTG |
| Pleurotus ostreatus alpha-like virus S1 | GCCAACGCGG TGTTAAGCTT TTTAGCC | ATACTCAGCC CTAGCCAAGG GCAAGTAG |
| Pleurotus ostreatus alpha-like virus S2 | CGACAG CTACGTCGCT CAACTACACG | GATCAGGTGC TGCTCTCGCT CTTTGAT |
| Pleurotus ostreatus alpha-like virus S3 | GTGGACTTAG CCGAATCCAT CAGGATG | CGGTTTGTTC TAGAAAGTTT CTCAATC |
| Pleurotus ostreatus ambivirus 2 S1 | GATATGGCAA ACAATCAGTC CCGCTG | AATGGGTCGA CTTGAATCCG GCAGGC |
| Pleurotus ostreatus ambivirus 2 S2 | GTAAG TAAATGTGGA ACTACCCGGT | TCTAGCTTCG CCTGTCTATA ACACGCGT |
| oyster mushroom spherical virus S1 | TATTGCACAT TGCGTTCCCC CTGCT | TCAAGACCGC CGCAACTGGA AAAGGTG |
| oyster mushroom spherical virus S2 | GTCTCCTGGT CGCACCTTGG CCCGT | ACCAAAACAAAACAAAACTCAAAATG |
| Pleurotus ostreatus ambivirus 3 S1 | AAGAGATGAT GCGTACTTTC CCGTG | ATTTTCTCCAGCGGTAGGGGAAGT |
| Pleurotus ostreatus ambivirus 3 S2 | ATACCAGGGG TTAAGCACGT GATAGG | CTTCCT CTTTTGTTTC GGGGTGCCT |
| Pleurotus ostreatus deltaflexivirus 3 S1 | GTGAGTCACT CTCTAAGGCC TATCTATC | CTATGAGCGC GGGAAAGGCT GAGTTTC |
| Pleurotus ostreatus deltaflexivirus 3 S2 | CCATTATGTG GCAAGAGTTC TTCCTGT | TCCGG ACCGGCAACC AAGACATAGT |
| Pleurotus ostreatus deltaflexivirus 3 S3 | AAGGAAACCT CTCCTTAGTT GCATTTCC | GAGAAAAGTT CAATTAACAT TGAACAAATC |
| Pleurotus ostreatus lentinuvirus 1 RNA1 S1 | CAAATTTTGA ATTTGTCATA TAAAAC | TTGAATTCAA GCATACTAA AACAGC |
| Pleurotus ostreatus lentinuvirus 1 RNA1 S2 | CAGAATGACC AATAGGAAGA TAACAAT | CATATACAA CTTAGTTAAT GGAAAATT |
| Pleurotus ostreatus lentinuvirus 1 RNA2 | ACACAAAGAT CGGAGTATAC AAATTTTG | CTACGAGAGT CAAGATGCCC AAGCCC |
| Primer pairs used for RACE | | |
| Virus name | 5' RACE (5'-3') | 3' RACE (5'-3') |
| Pleurotus ostreatus deltaflexivirus 2 | GATTACGCCAAGCTTGGGAGGGAGTAATGAGCGTG GAAGCCG | GATTACGCCAAGCTT GCCCGTGTCC GTGTCTGGTT GACGTTC |
| Pleurotus ostreatus deltaflexivirus 3 | GATTACGCCAAGCTTGGGGTAATGAGTGTGGAGGCGGTCAACC | GATTACGCCAAGCTT  GATCGCTCGT CGATCGGTTA CCATAGG |
| Pleurotus ostreatus lentinuvirus 1 | RNA1:GATTACGCCAAGCTT CA TCACAGAAGC CATGATGGGA CCAGC  RNA2:GATTACGCCAAGCTTGTACCGA GGAACTGTGG CTGAGGAGTG | RNA1:GATTACGCCAAGCTTGCCTGGGGATGATCTGACTACGATAGAC  RNA2:GATTACGCCAAGCTTGGTGCTCTGT ACATGCCGGA AGTGGGC |
| Pleurotus ostreatus alpha-like virus | GATTACGCCAAGCTTGCGA CGTCCAAGCG CGCCTCAATG TTGC | GATTACGCCAAGCTTCCGGTCCTGG TATCTTTGGA CGTGCGG |
| Pleurotus ostreatus ambivirus 2 | GATTACGCCAAGCTT CC CGCTTCGCCC ATAACACCTA TGACCCC | GATTACGCCAAGCTTGCTGGGCCAT GTTTTGGCAT AGGGTACG |
| Pleurotus ostreatus ambivirus 3 | GATTACGCCAAGCTT TGC CCCATCGTTT CTCGACTCAA CCGG | GATTACGCCAAGCTT TCCTGGAAGG GACGACCCAT AAGCTGC |
| oyster mushroom spherical virus | GATTACGCCAAGCTTGGTGC CGATAAGGCG TGAACCACCG | GATTACGCCAAGCTTGATGTCCTTC TCCCAGCCCG GTCCTTG |

*These primer pairs were used for RT-PCR identification.
