# Supplementary material for: Deep sequencing of single Pleurotus ostreatus strain revealed mixed infection of seven RNA viruses
Source: BMC Genomics. 2026 Jul 17;27:843. doi: 10.1186/s12864-026-13166-z (PMC13660232; doi:10.1186/s12864-026-13166-z)
Supplement: Supplementary file 2 [file 12864_2026_13166_MOESM2_ESM.doc]

**Supplemental Figures**

**M 1 2 3 4 5	6 7**

Figure S1 Agarose gel electrophoresis analysis of the RT-PCR amplicons of candidate viruses. M: DL2000 marker, Lanes1-7, oyster mushroom spherical virus, pleurotus ostreatus deltaflexivirus 2, pleurotus ostreatus deltaflexivirus 3, pleurotus ostreatus alpha-like virus, pleurotus ostreatus ambivirus 2, pleurotus ostreatus ambivirus 3, pleurotus ostreatus lentinuvirus 1

**M	1	2	M	3	4	5	M	6	7	M	8**

**M	1	2	3	4	M	5	6	M	7	8**

**E**

**F**

**G**

Figure S2 Agarose gel electrophoresis analysis of the rapid amplification of cDNA ends of identified viruses and the target bands were labeled with red boxes. M: DL2000 marker; lanes 1-8 in upper panel, 5′ RACE of pleurotus ostreatus deltaflexivirus 2, pleurotus ostreatus deltaflexivirus 3, pleurotus ostreatus lentinuvirus 1 RNA2, pleurotus ostreatus lentinuvirus 1 RNA1, pleurotus ostreatus alpha-like virus, pleurotus ostreatus ambivirus 2, pleurotus ostreatus ambivirus 3 and oyster mushroom spherical virus; lanes 1-8 in lower panel, 3′ RACE of pleurotus ostreatus deltaflexivirus 2, pleurotus ostreatus deltaflexivirus 3, pleurotus ostreatus ambivirus 2, pleurotus ostreatus ambivirus 3,pleurotus ostreatus lentinuvirus 1 RNA2, pleurotus ostreatus lentinuvirus 1 RNA1,pleurotus ostreatus alpha-like virus and oyster mushroom spherical virus.

Figure S3 The predicted A/U rich stem-loop structure in the noncoding [intergenic region](https://www.sciencedirect.com/topics/agricultural-and-biological-sciences/intergenic-region) of of pleurotus ostreatus lentinuvirus 1-SBX RNA2

Figure S4 Coinfection pattern of the collected and isolated strains from different locations.
